# Prince of Wales's Hospital Fund for London

**Published:** 1898-02-05

**Authors:** 


### PRINCE OF WALES'S HOSPITAL FUND FOR LONDON.

### FIRST ANNUAL MEETING.

The first annual meeting of the Council of the Prince of Wales's Hospital Fund for London was held yesterday afternoon at Marlborough House, H.R.H. the Prince of Wales in the chair. There were also present the Duke of Norfolk, the Bishop of London, the Bishop of Rochester, the Rev. J. Guinness Rogers, D.D., the Rev. T. Bowman Stephenson, D.D., the Chief Rabbi, Lord Rowton, Lord Rothschild, Lord Iveagh, Lord Farquhar, Dr. Collins (Chairman of the London County Council), Mr. Hugh C. Smith (Governor of the Bank of England), Sir Samuel Wilks, Bart. (President of the Royal College of Physicians), Sir William MacCormac, Bart. (President of the Royal College of Surgeons), the Right Hon. C. Stuart Wortley, M.P., Q.C., Sir Henry Burdett, K.C.B., Mr. Sydney Buxton, Mr. Albert G. Sandeman, Mr. William Latham, Q.C., Sir Savile Crossley, Bart. (hon. secretary), and Mr. J. G. Craggs (hon. assistant secretary).

The minutes of the previous meeting having been read and confirmed, Sir Savile Crossley (hon. secretary), stated that letters regretting their inability to attend had been received from the Duke of Westminster, the Earl of Strafford, Cardinal Vaughan, the Lord Mayor, Lord Lister, Lord Reay, Mr. John Aird, M.P., and Mr. E. A. Hambro.

Mr. Hugh C. Smith presented the report of the Executive Committee, in which it was stated that the distribution of grants to the hospitals had been effected in accordance with the directions of the General Council given on December 20th, 1897.

H.R.H. the Prince of Wales moved the adoption of the report of the Executive Committee.

This was seconded by Lord Rothschild, and carried.

The annual report (given on page 3) and accounts were presented to the meeting.

Sir Savile Crossley pointed out that the accounts attached to the report were draft accounts, and, therefore, subject to revision in course of the audit.

H.R.H. the Prince of Wales moved that the annual report and accounts (subject to audit) be received and adopted by the Council.

Lord Rothschild seconded, and the motion was agreed to unanimously.

The Bishop of London said that one of the most useful elements in the work done by the Committee throughout the year had been the sale of Jubilee Hospital Stamps, and it was hoped that a considerable portion, at any rate, of the amount received this year from that source might in future be counted upon as an annual subscription. The provision of the albums and stamps had proved a ready means for collecting subscriptions, and it was believed that there were a number of people who would take advantage of the opportunity thus afforded them of becoming subscribers to the hospitals. He had therefore much pleasure in moving the following resolution: "That the best thanks of the Council are hereby given to those who have successfully arranged the sale of the Jubilee Hospital Stamps, and that Sir Henry Burdett, in conjunction with the Executive Committee, be requested to undertake the arrangements connected with the sale of the stamps for the present year, and be authorised to act on behalf of the Council in this respect."

The Duke of Norfolk had very great pleasure in seconding that motion. He was sure that those who were acquainted with the subject knew what extreme trouble Sir Henry Burdett had taken in the matter, and he had to convey to him the thanks of all those interested in the Fund for his efforts. Above all, however, the thanks of the Council in this matter were due to His Royal Highness, who had given not only the all-powerful support of his encouragement, but his active co-operation to bring about the great success which these stamps had proved to be.

H.R.H. the Prince of Wales: Before putting that resolution I must endorse what fell from the lips of the Bishop of London and the Duke of Norfolk with regard to the unwearying labour and attention Sir Henry Burdett has given to this matter, which has been so far successful, and will, I hope, continue to be so. I hope also that, in accordance with the unanimous wish of the Council, he will continue, in conjunction with the Executive Committee, to undertake these duties, so that the Hospital Stamps may become an annual source of revenue.

The motion was then put to the meeting, and carried unanimously.

Sir William MacCormac said that in the unavoidable absence of Lord Lister, he had been asked to propose the next resolution. The Prince of Wales, at the last meeting of the Council, had clearly indicated the way in which the moneys received by the Fund were to be distributed this year, but those familiar with hospitals knew that their conditions changed from time to time, and that a most intimate knowledge of their requirements and needs, and the good they might do, was necessary in order properly to allocate the funds in the future. He therefore had much pleasure in proposing the following resolution: "That the

Council considers it essential that a Committee of Enquiry be at once appointed to visit personally and examine the various hospitals of the Metropolis, with a view to ascertaining their present condition, needs, and merits, for the allocation of the funds received during 1898; and that the Executive Committee be authorised to take the necessary steps forthwith."

The Rev. J. Guinness Rogers, D.D., in seconding, ventured to think that it was on the Committee of Enquiry that the successful working and efficiency of the Fund would depend. At the present time the air was full of objections from all kinds of objectors. A number of people seemed simply to live for the sake of objecting, and the only way in which the Fund could possibly meet the objectors was to take a step like that proposed, which would place the whole matter of the management of the hospitals entirely outside the range of that miserable kind of controversy which confronted every good movement.

H.R.H. the Prince of Wales said: This is perhaps, the most important matter which we have, before us to-day. I hope that the committee of inquiry into the state of all the hospitals will be confined to the members of the Council. I do not think that we need go outside the Council to find members for that committee, as we are competent ourselves to make our own inquiries. It will take time, of course, and it will have to be done delicately; but I do not think it possible for us to keep to our original idea, *i.e.*, to assist the most deserving hospital only, unless it is made quite clear to us which hospitals are really deserving and which are managed in a proper way. I certainly am of opinion that all these inquiries should be kept private, as it would not be fair to the hospitals nor would it be fair to ourselves to use confidential reports in order to represent them to the public as being in an unsatisfactory condition. We might, however, offer advice in cases were it was necessary, and if the hospitals agreed to accept our advice we might then consider the question of a grant.

Lord Rothschild said he was sure they all fully appreciated the remarks made by his Royal Highness as to confining the committee to members of the Council. There was no limit to the number of members on the Council, and it rested with His Royal Highness, on the advice of that Council, to add to its members those whom he might think fit. In his opinion they had reason to congratulate themselves on the success of the undertaking, even though they had not received as much money as was expected. They had aroused in the Metropolis an interest in all the hospitals which must be beneficial to them in the future. At the same time they had aroused a certain amount of hostility because they had been endowed with virtues which they did not possess, and which they had never intended to possess. It was said that they wished to get into their hands the management of the hospitals, and they were supposed to be anxious to do away with outdoor relief and letters. He was sure it had never entered the minds of those present to interfere with the hospitals. All they wanted was to make sure that if they distributed money it should be distributed to the hospitals which they thought were the best managed.

The resolution was put to the meeting, and carried unanimously.

H.R.H. the Prince of Wales: All that remains now for me to do is to convey my most sincere thanks to the members of the Council generally, and to the Executive Committee in particular, for the great assistance they have given me in carrying out this scheme, which I started a year ago, and for giving me so much of their valuable time. I hope they will all consent to serve again and to assist me for another year. The Fund is now well started. It depends very much on the subscriptions we receive from the public what we may do at the end of this year. Still I hope that if we go on as we have done so far, the public will see that we are a sound body, and that we are using our utmost endeavours to carry out what was our original object, namely, to maintain the great hospitals of this Metropolis, to do all we can to assist those which are in need of funds, and to prove a lasting memorial of the Sixtieth Anniversary of the Reign of the Queen.

The meeting then terminated.

### PRINCE OF WALES'S HOSPITAL FUND FOR LONDON.

### REPORT OF COUNCIL FOR 1897.

This, the first annual report of the Fund, would be incomplete without some account of its origin. The simplest and clearest statement is that made by His Royal Highness the President in his letter to the public of February 5th, 1897 (see The Hospital for February 13th, 1897).

The Council formed to assist His Royal Highness in carrying out the scheme was originally composed of those who were invited to Marlborough House on January 21st, 1897, with the addition, from time to time, of such persons as seemed qualified to make the Council still more representative in its nature. This Council in its turn appointed an Executive Committee to make arrangements requisite to the success of the scheme. Other committees were appointed for various sections of the work, and a strictly limited number of appeals were issued.

It was essential to the success of the Fund that no encroachment should be made on the ground covered by the Hospital Sunday and Saturday Funds. Any danger of this kind was avoided by agreement, leaving the Sunday Fund to seek the advocacy of the pulpit, and the Saturday Fund the collections made in workshops, unless they were clearly stated to be sent to the Prince's Fund in honour of Her Majesty's Jubilee.

A great impetus was given when His Royal Highness the President announced that his appeal had the sanction of the Queen.

The Council desire to express their indebtedness to the metropolitan Press, and especially to those newspapers which opened subscription lists in their columns, or which co-operated in other ways to interest Londoners in the Fund, and so gave the movement an excellent start.

It would be invidious to select any subscription for special mention, but a list of contributors will be found in the report. It may here suffice to say that the individual donations run from 6d to £12,500, and the subscriptions from 1s. to £1,000 a year each.

Two conferences of representatives of the clergy and ministers of all religious bodies assembled at London House at the invitation of the Bishop of London, who presided on both occasions. It was unanimously resolved at the first conference, held in February, 1897, "That the members believe that the ministers of the various denominations will be able and willing to co-operate with the Executive Committee of the Prince of Wales's Hospital Fund on the basis of the understanding already referred to."

In accordance with a later recommendation of these conferences, the subscription books and stamp albums issued by the Fund have been brought before the attention of the clergy and ministers throughout London, with a request that they will consider the possibility of finding some one connected with each congregation who will bring before the people this method of making small subscriptions. The Council venture to hope that they will have the cordial co-operation of the leaders in spiritual work throughout London in their endeavour to interest the masses of the people to become small subscribers to the hospitals through the Fund.

Soon after the Fund was established, district committees were organised in Paddington by Mr. John Aird, M.P., and in Marylebone by Sir Horace Farquhar, Bart., M.P., being the members for the respective divisions in question. So far, these are the only metropolitan districts which have thus organised branches; but a general desire has been expressed that the district committees already appointed should be kept together for the purpose of getting in the income they have been promised for the Prince's Fund, and that they may be called together as occasion may require. It is hoped that the success so far achieved may encourage other districts to establish committees with the same object.

The sale of the Jubilee Procession Programme, the publication of which was made possible by the generous co-operation of the *Daily Graphic*, realised upwards of £2,000; the Hospital Stamps, which were engraved by Messrs. De la Rue and Co. free of cost, have already produced a considerable sum, and it is hoped that by issuing different stamps each year numerous contributions will be secured for the Fund.

A memorial has been presented to His Royal Highness the President, from those in favour of the establishment of a Central Hospital Board for London, urging its formation. The Council, however, with His Royal Highness's approval, resolved: "That this Council hereby approves and adopts the resolution passed by the Executive Committee on November 9th, 1897, that, in view of the controversial character of the proposition of the Charity Organisation Society to establish a Central Hospital Board for London, it is inexpedient that the Prince of Wales's Hospital Fund for London be associated with the movement."

A special committee was appointed under the chairmanship of Lord Lister to consider and report as to which of the leading hospitals in the metropolis should receive assistance from the Fund during 1897; and the proportions in which the money should be granted. The substance of the report of this special committee follows.

Careful and complete inquiries were made into the particular and local needs of those hospitals in which there was a permanent occupancy of 100 or more beds on an average of three years. These institutions include, though not exclusively, all the metropolitan hospitals which have medical schools attached to them; and it was decided to devote the income of the Fund (£22,050) to annual grants to such of them as specially needed help for the purposes of opening closed wards, and for other pressing needs. Those hospitals which receive such annual grants are thirteen in number. Besides thus employing the income of the Fund, the Council resolved that a sum of about £34,000 received from the sale of the Hospital Stamps should be given this year to the hospitals generally in the form of special donations in honour of the Diamond Jubilee of Her Majesty the Queen, and in accordance with the Hospital Sunday Fund awards. The institutions receiving special donations on this basis included twenty-eight general hospitals, five chest hospitals, fourteen children's hospitals, six lying-in hospitals, six women's hospitals, and twenty-five other special hospitals. It may be anticipated that in all these hospitals the considerable sums they thus receive will assist them in carrying out greater efficiency and in increasing their usefulness; while, further, no less than about 180 beds in wards now closed to the poor will henceforth be available for them. The Council are agreed that it is extremely desirable that the wants and merits of all the metropolitan hospitals should be investigated, and in future years it is their intention to see that this shall be done. It was not possible to do it completely, however, in the first year of the Fund's working, and that is why, having regard to the terms of the Prince of Wales's original letter to the public, the Hospital Sunday Fund basis was resorted to in determining the amounts of the special donations.

The Council believe that the way in which the moneys have been distributed has met with general approval, and they venture to hope that it will lead the public to show increasing confidence in the Fund during 1898, so that not only may the assistance already given to the leading hospitals be continued, but that similar aid may be extended to every hospital throughout the metropolis which is found on investigation to be worthy of a grant.

The total sum paid into the credit of the Fund during the eleven months ended December 31st, 1897, has been £227,553 12s. 5d. Of this amount there has been given in annual subscriptions, chiefly in the form of bankers' orders, £21,535 6s. 11d.

There is reason to expect that many of the donors of 1897 will renew their donations each year.

After providing the sum of £56,826 5s., which has been distributed amongst the hospitals during 1897, there remained a sum of £167,022 19s. 8d. for investment. The most strenuous endeavours have been made to reduce the expenses to the lowest possible sum. The total expenditure under all heads, including salaries, office and other expenses, advertisements, &c., amounts to £3,704 7s. 9d. It follows that the whole of the £227,553 12s. 5d., being the total sum received by the Fund, has been raised at a cost of £1 12s. 6d. per cent.

From first to last His Royal Highness the President has been untiring in his interest in the work of the Fund. He continues to devote himself to every detail, and has freely given his time to the development of a movement which he feels to be of very real importance to the welfare of those who live in the metropolis of the empire.

### REPORT OF THE SPECIAL COMMITTEE ON DISTRIBUTION.

The committe unanimously present the following report. We have proceeded on the supposition that in all probability grants will be made for this year only from the Prince of Wales's Fund to all hospitals which receive awards from the Hospital Sunday Fund, and in approximately the same amounts.

Assuming such contributions to be made, they may be regarded as special grants for this year, leaving the present income of the Prince's Fund to be disposed of in the manner which may seem best calculated to benefit the hospitals of the metropolis.

In accordance with views expressed and adopted in the Executive Committee, it has appeared to us that the income money of the Fund cannot be better spent than in aiding, in proportion to their needs and usefulness, the most important of the hospitals. A fair indication of those institutions which may be regarded in this light is the permanent occupancy of 100 or more beds. This test will include, though not exclusively, all the hospitals having medical schools. And it may be remarked that the existence of a school in connection with a hospital gives it a strong claim to support; not only because a medical school in itself confers immense benefits on the community, but also because the public criticism to which the officers of such a hospital are perpetually subjected ensures that the treatment of the patients is conducted in accordance with the best existing knowledge.

The hospitals in the metropolis having 100 or more beds permanently occupied on the average of the three years, 1894 to 1896, are 17 in number, and are as follows:—

| | |
|---|---|
| Charing Cross Hospital | (146) |
| Childrens' Hospital, Great Ormond Street | (146) |
| City of London Hospital for Diseases of the Chest ... ... ... ... | (106) |
| Guy's Hospital ... ... | (423) |
| Hospital for Consumption, Brompton ... | (246) |
| King's College Hospital | (180) |
| The London Hospital... | (634) |
| Middlesex Hospital ... | (275) |
| National Hospital for the Paralysed, &c. ... | (165) |
| Royal Free Hospital ... | (119) |
| St. Bartholomew's Hospital ... ... ... ... | — |
| St. George's Hospital... | (299) |
| St. Mary's Hospital ... | (247) |
| St. Thomas's Hospital | (375) |
| Seamen's Hospital ... | (203) |
| University College Hospital ... ... ... ... | (185) |
| Westminster Hospital | (165) |

The average number of beds occupied daily per annum on the average of the three years, 1894 to 1896, have been added in parentheses.

With the view of ascertaining the relative needs of these institutions, the committee addressed a letter to the chairman of each of them, except St. Bartholomew's, requesting information under certain heads. Among these heads we include that of vacant beds. To open wards closed for want of funds is certainly a most important object: and on the understanding that we are dealing with the income of the Fund, and that a sum given to any hospital this year for the purpose under consideration will probable be continued in future years, and so may be regarded as a grant of so much per annum, we have recommended that considerable sums should be so employed. It may seem superfluous to point out that it would be mere mockery to invite any institution to undertake the opening of a closed ward by making a grant to it this year, unless there were reasonable prospect that a similar provision could be made in the future.

Of course it will always be in the power of the Council to modify the amounts in future years either by way of increase or diminution, in accordance with the varying needs of the various hospitals. But the allocation of any portions of the Fund to the extremely important object of opening closed wards, must, in our opinion, be done on the understanding that it will be on a more or less permanent footing.

Supposing our recommendations in this respect to be acted on, the result would be the immediate and permanent occupying, in wards now closed, of 169 beds in general hospitals and 16 in children's hospitals, making a total of 185 beds. This would in itself constitute an immense boon to the suffering poor of London.

Besides making this inquiry by letter we have endeavoured to ascertain the financial condition of the various hospitals by taking out the value of the capital funds of each institution based on the actual income from general investments, which we have capitalised at 3 per cent., and the income derived from rentals of house property, &c., which we have capitalised at 5 per cent. The figures for the years 1886 and 1896 have been checked and certified by Messrs. Craggs, Turketine, and Co., chartered accountants. St. Bartholomew's Hospital is not included in the certificate, because it is well endowed, the whole of its revenue being derived from invested property.

We have further to point out in this connection that the condition of the fabric of some of the older hospitals imperatively demands a capital expenditure upon the buildings and appliances, while a further sum ought to be expended in renewing the furniture and internal fittings, so as to bring the hospitals up to modern requirements and modern efficiency. In the opinion of the committee there should be a mean fixed point arrived at by each hospital committee, which should represent the average amount in pounds sterling at which the reserve fund of each hospital should stand, in order to secure it from any temporary loss or decreasing income due to special causes. It has been suggested, and is held by those who have given a great deal of time to the consideration of the question, that any voluntary hospital which has reserves amounting to a sum equal to five years' gross annual expenditure, has attained a position of financial soundness which should enable its governing body to devote any available portion of the remaining capital, if and when necessary, to defray the expenditure upon new buildings, or the renewal of old buildings, drainage, sanitary improvements, refurnishing, and so forth.

The Committee are anxious that each of the great hospitals shall be modernised and improved, when necessary, by the expenditure of money out of capital reserve funds upon the renovation of old buildings, the improvement of their sanitary condition, and the renewal of furniture and fittings throughout these establishments.

It is felt that a definite increase in the income of a hospital through the instrumentality of the Prince of Wales's Fund must encourage the committee of each institution to take in hand the work of renovation and improvement, and to defray the cost of it out of the reserve fund. By this system the financial condition of each of the principal hospitals would be strengthened in accordance with the wishes expressed in His Royal Highness's letter dated February 5th, 1897.

The sums which we have recommended to be specially allocated to the principal hospitals amount in all to £22,050. This, added to what is likely to be given to all hospitals on the basis of the Hospital Sunday Fund (£34,776 5s.), makes the sum distributed this year £56,826 5s.

(Signed) Lister, Chairman.

December 14th, 1897.

### PRINCE OF WALES'S HOSPITAL FUND FOR LONDON.

LIST OF AWARDS FOR THE YEAR 1897.

| NAME OF HOSPITAL. | Annual Grants. | | | Special Donations for this Year only in honour of Her Majesty's Diamond Jubilee. | | |
|---|---|---|---|---|---|---|
| **METROPOLITAN HOSPITALS.** | | | | | | |
| HOSPITALS WITH 100 BEDS IN CONSTANT OCCUPATION. | £ | s. | d. | £ | s. | d. |
| Brompton Hospital for Consumption | — | | | 1,391 | 5 | 0 |
| Charing Cross Hospital | 1,000 | 0 | 0 | 1,006 | 5 | 0 |
| City of London Hospital for Diseases of the Chest | — | | | 927 | 10 | 0 |
| Guy's Hospital | 6,600 | 0 | 0 | 1,312 | 10 | 0 |
| Hospital for Sick Children, Great Ormond Street, W.C. | 500 | 0 | 0 | 700 | 0 | 0 |
| King's College Hospital | 1,000 | 0 | 0 | 1,400 | 0 | 0 |
| London Hospital | 5,000 | 0 | 0 | 3,937 | 10 | 0 |
| Middlesex Hospital | 1,000 | 0 | 0 | 1,925 | 0 | 0 |
| National Hospital for the Paralysed and Epileptic | 750 | 0 | 0 | 752 | 10 | 0 |
| Royal Free Hospital | 750 | 0 | 0 | 831 | 5 | 0 |
| St. George's Hospital | — | | | 1,356 | 5 | 0 |
| St. Mary's Hospital | 1,000 | 0 | 0 | 1,706 | 5 | 0 |
| St. Thomas's Hospital | 1,800 | 0 | 0 | — | | |
| Seamen's Hospital Society | 500 | 0 | 0 | 918 | 15 | 0 |
| University College Hospital | 1,400 | 0 | 0 | 1,181 | 5 | 0 |
| Westminster Hospital | 750 | 0 | 0 | 1 093 | 15 | 0 |
| OTHER GENERAL. | | | | | | |
| French Hospital | — | | | 323 | 15 | 0 |
| German Hospital | — | | | 393 | 15 | 0 |
| Great Northern Central Hospital | — | | | 481 | 5 | 0 |
| Hampstead Hospital | — | | | 131 | 5 | 0 |
| Italian Hospital | — | | | 61 | 5 | 0 |
| London Homœopathic Hospital | — | | | 245 | 0 | 0 |
| London Temperance Hospital | — | | | 647 | 10 | 0 |
| Metropolitan Hospital | — | | | 481 | 5 | 0 |
| Mildmay Mission Hospital | — | | | 262 | 10 | 0 |
| Miller Hospital and Royal Kent Dispensary | — | | | 236 | 5 | 0 |
| North-West London Hospital | — | | | 350 | 0 | 0 |
| Phillips' Memorial Homœopathic Hospital | — | | | 30 | 12 | 6 |
| Poplar Hospital | — | | | 402 | 10 | 0 |
| SS. John and Elizabeth Hospital | — | | | 87 | 10 | 0 |
| West Ham Hospital | — | | | 280 | 0 | 0 |
| West London Hospital | — | | | 525 | 0 | 0 |
| OTHER SPECIAL. | | | | | | |
| North London Consumption Hospital | — | | | 262 | 10 | 0 |
| Royal Hospital for Diseases of the Chest | — | | | 350 | 0 | 0 |
| Royal National Hospital for Consumption (Ventnor) | — | | | 262 | 10 | 0 |
| Alexander Hospital for Hip Disease | — | | | 148 | 15 | 0 |
| Barnet Home Hospital | — | | | 35 | 0 | 0 |
| Belgrave Hospital for Children | — | | | 122 | 10 | 0 |
| Cheyne Hospital for Incurable Children | — | | | 122 | 10 | 0 |
| East London Hospital for Children | — | | | 393 | 15 | 0 |
| Evelina Hospital for Sick Children | — | | | 393 | 15 | 0 |
| Home for Incurable Children, Maida Vale, W. | — | | | 61 | 5 | 0 |
| Home for Sick Children, Sydenham, S.E. | — | | | 122 | 10 | 0 |
| North-Eastern Hospital for Children | — | | | 262 | 10 | 0 |
| Paddington Green Hospital for Children | — | | | 148 | 15 | 0 |
| Victoria Hospital for Children | — | | | 481 | 5 | 0 |
| Victoria Home, Margate | — | | | 26 | 5 | 0 |
| "The Vine," Sevenoaks | — | | | 26 | 5 | 0 |
| British Lying-in Hospital | — | | | 26 | 5 | 0 |
| City of London Lying-in Hospital | — | | | 65 | 12 | 6 |
| Clapham Maternity Hospital | — | | | 30 | 12 | 6 |
| East-End Mothers' Home | — | | | 61 | 5 | 0 |
| General Lying-in Hospital | — | | | 52 | 10 | 0 |
| Queen Charlotte's Lying-in Hospital | — | | | 201 | 5 | 0 |
| Chelsea Hospital for Women | — | | | 306 | 5 | 0 |
| Hospital for Women, Soho Square, W. | — | | | 306 | 5 | 0 |
| Grosvenor Hospital for Women and Children | — | | | 43 | 15 | 0 |
| New Hospital for Women | — | | | 192 | 10 | 0 |
| Royal Hospital for Children and Women | — | | | 218 | 15 | 0 |
| Samaritan Free Hospital | — | | | 490 | 0 | 0 |
| Cancer Hospital, Brompton, S.W. | — | | | 568 | 15 | 0 |
| London Fever Hospital | — | | | 875 | 0 | 0 |
| Gordon Hospital for Fistula, Vauxhall Bridge Road, S.W. | — | | | 36 | 15 | 0 |
| St. Mark's Hospital for Fistula, City Road, E.C. | — | | | 52 | 10 | 0 |
| National Hospital for Diseases of the Heart and Paralysis, Soho Square, W. | — | | | 52 | 10 | 0 |

OTHER SPECIAL HOSPITALS—(*continued.*)

| NAME OF HOSPITAL. | Annual Grants. | Special Donations for this Year only in honour of Her Majesty's Diamond Jubilee. |
|---|---|---|
| | £ s. d. | £ s. d. |
| Female Lock Hospital, Harrow Road, W. ... ... ... ... ... ... | — | 122 10 0 |
| Hospital for Epilepsy, Paralysis, and other Diseases of the Nervous System, Regent's Park, N.W. ... ... ... ... ... ... ... ... | — | 56 17 6 |
| West-End Hospital for Diseases of the Nervous System ... ... ... ... | — | 96 5 0 |
| Central London Ophthalmic Hospital, Gray's Inn Road, W.C. ... ... ... | — | 26 5 0 |
| Royal Eye Hospital, St. George's Circus, S.E. ... ... ... ... ... ... | — | 166 5 0 |
| Royal London Ophthalmic Hospital, Moorfields, E.C.... ... ... ... ... ... | — | 490 0 0 |
| Royal Westminster Ophthalmic Hospital, Charing Cross, W.C. ... ... ... | — | 87 10 0 |
| Western Ophthalmic Hospital, Marylebone Road, W.... ... ... ... ... | — | 39 7 6 |
| City Orthopædic Hospital, Hatton Garden, E.C. ... ... ... ... ... | — | 65 12 6 |
| National Orthopædic Hospital, Great Portland Street, W. ... ... ... ... | — | 70 0 0 |
| Royal Orthopædic Hospital, Oxford Street, W.... ... ... ... ... ... | — | 61 5 0 |
| Royal Sea-Bathing Infirmary, Margate ... ... ... ... ... ... ... | — | 297 10 0 |
| St. Peter's Hospital for Stone, Covent Garden, W.C. ... ... ... .. ... | — | 26 5 0 |
| Central London Throat and Ear Hospital, Gray's Inn Road, W.C. ... ... ... | — | 35 0 0 |
| Royal Ear Hospital, Frith Street, W. ... ... ... ... ... ... ... | — | 7 17 6 |
| Dental Hospital of London, Leicester Square, W.C. ... ... ... ... ... | — | 109 7 6 |
| National Dental Hospital, 149, Great Portland Street, W. ... ... ... ... | — | 105 0 0 |
| **COTTAGE HOSPITALS.** | | |
| Beckenham Cottage Hospital ... ... ... ... ... ... ... ... ... | — | 45 15 0 |
| Blackheath and Charlton Cottage Hospital ... ... ... ... ... ... ... | — | 61 5 0 |
| Bromley, Kent, Cottage Hospital ... ... ... ... ... ... ... ... | — | 72 12 6 |
| Chislehurst, Sidcup, and Cray Valley Cottage Hospital ... ... ... ... | — | 39 7 6 |
| Eltham Cottage Hospital ... ... ... ... ... ... ... ... ... ... | — | 28 17 6 |
| Enfield Cottage Hospital ... ... ... ... ... ... ... ... ... ... | — | 39 7 6 |
| Epsom and Ewell Cottage Hospital ... ... ... ... ... ... ... ... | — | 61 5 0 |
| Hounslow Cottage Hospital... ... ... ... ... ... ... ... ... ... | — | 17 10 0 |
| Mildmay Cottage Hospital ... ... ... ... ... ... ... ... ... ... | — | 61 5 0 |
| Reigate and Redhill Cottage Hospital ... ... ... ... ... ... ... ... | — | 52 10 0 |
| Sidcup Cottage Hospital ... ... ... ... ... ... ... ... ... ... | — | 30 12 6 |
| Wimbledon Cottage Hospital ... ... ... ... ... ... ... ... ... | — | 35 0 0 |
| Woolwich and Plumstead Cottage Hospital ... ... ... ... ... ... ... | — | 30 12 6 |
| **INSTITUTIONS.** | | |
| Establishment for Gentlewomen, Harley Street... ... ... ... ... ... | — | 105 0 0 |
| Invalid Asylum, Stoke Newington ... ... ... ... ... ... ... ... | — | 52 10 0 |
| **SUMMARY.** | | |
| HOSPITALS WITH 100 BEDS IN CONSTANT OCCUPATION ... ... ... ... ... | 22,050 0 0 | 20,440 0 0 |
| OTHER GENERAL HOSPITALS ... ... ... ... ... ... ... ... ... ... | — | 4,939 7 6 |
| OTHER SPECIAL HOSPITALS ... ... ... ... ... ... ... ... ... ... | — | 8,663 7 6 |
| COTTAGE HOSPITALS ... ... ... ... ... ... ... ... ... ... | — | 576 0 0 |
| INSTITUTIONS ... ... ... ... ... ... ... ... ... ... ... | — | 157 10 0 |
| TOTAL ... ... ... ... ... | £22 050 0 0 | £34,776 5 0 |

### UNIVERSITY COLLEGE HOSPITAL.

THE Duke of BEDFORD, the President of the Hospital, presided at the Festival Dinner of the University College Hospital, which was held at the Whitehall Rooms, on Wednesday, the 2nd inst. Amongst those present were: The Duchess of Bedford, Lord and Lady Reay, Sir Douglas Galton, Mr. Benjamin L. Cohen, M.P., Mr. R. G. Webster, M.P., Mr. Christopher Heath, Dr. Hare, Dr. Sydney Ringer, Mr. and Mrs. Arthur Lucas, Mr. and Mrs. Henry Lucas, and Mr. Augustus and Miss Prevost.

In proposing the toast of "The Prince and Princess of Wales and the rest of the Royal Family," the CHAIRMAN said he felt sure that the Prince's health would be drunk that evening with extra enthusiasm, seeing that they were the Prince's almoners, as through the Prince of Wales' Fund they had been enabled to open 25 of the 50 beds which, for financial reasons, they had been obliged to close. (Applause.)

Lord REAY and Mr. BENJAMIN L. COHEN, M.P., responded for "The Houses of Lords and Commons," which was proposed by Sir DOUGLAS GALTON.

In proposing the toast of the evening, the CHAIRMAN said that when he took up the presidency of the hospital he expected that there was work—some of it unpleasant work—ahead of him. It was unpleasant to have beds closed and to know that there had been a serious falling off in legacies. What was the cause of the falling off? Some would say that the generous people of this country were becoming sceptical as to the economical working of hospitals, and all who had the interests of hospitals at heart would agree that the sooner the question of autonomy or federation was decided the better. He would express no opinion save this, that if—and this was a big "if"—a Central Hospital Board could be formed which would improve the finances and improve the efficiency of the hospitals he was for such a Board and would welcome its control. (Hear, hear.) He could not at present pass over in silence the hospital's unsatisfactory financial position. What would that dreadful "official receiver," public opinion, say when it looked into the accounts of the hospital, "Living beyond their means and gambling on the off-chance of great benefactions from the wealthy." Referring to the Prince of Wales' Fund, he said that that fund had left no stone unturned to find the necessary money for the hospitals of London, and through its means 25 of the hospital's deserted beds would be reopened—(applause)—and he appealed to those present to help ito find money to open the remaining 25 beds. (Loud applause.)

The collection announced by the secretary as the result of the dinner was £63 1s. in annual subscriptions, and £2,941 10s. donations.

